# IL-10R Blockade during Chronic Schistosomiasis Mansoni Results in the Loss of B Cells from the Liver and the Development of Severe Pulmonary Disease

**DOI:** 10.1371/journal.ppat.1002490

**Published:** 2012-01-26

**Authors:** Keke C. Fairfax, Eyal Amiel, Irah L. King, Tori C. Freitas, Markus Mohrs, Edward J. Pearce

**Affiliations:** 1 Department of Pathology and Immunology, Washington University School of Medicine, St. Louis, Missouri, United States of America; 2 Trudeau Institute, Saranac Lake, New York, United States of America; NIAID/NIH, United States of America

## Abstract

In schistosomiasis patients, parasite eggs trapped in hepatic sinusoids become foci for CD4^+^ T cell-orchestrated granulomatous cellular infiltrates. Since the immune response is unable to clear the infection, the liver is subjected to ongoing cycles of focal inflammation and healing that lead to vascular obstruction and tissue fibrosis. This is mitigated by regulatory mechanisms that develop over time and which minimize the inflammatory response to newly deposited eggs. Exploring changes in the hepatic inflammatory infiltrate over time in infected mice, we found an accumulation of schistosome egg antigen-specific IgG1-secreting plasma cells during chronic infection. This population was significantly diminished by blockade of the receptor for IL-10, a cytokine implicated in plasma cell development. Strikingly, IL-10R blockade precipitated the development of portal hypertension and the accumulation of parasite eggs in the lungs and heart. This did not reflect more aggressive Th2 cell responsiveness, increased hepatic fibrosis, or the emergence of Th1 or Th17 responses. Rather, a role for antibody in the prevention of severe disease was suggested by the finding that pulmonary involvement was also apparent in mice unable to secrete class switched antibody. A major effect of anti-IL-10R treatment was the loss of a myeloid population that stained positively for surface IgG1, and which exhibited characteristics of regulatory/anti-inflammatory macrophages. This finding suggests that antibody may promote protective effects within the liver through local interactions with macrophages. In summary, our data describe a role for IL-10-dependent B cell responses in the regulation of tissue damage during a chronic helminth infection.

## Author Summary

Schistosomiasis is a chronic disease that affects approximately 200 million people. Immune modulation is a hallmark of chronic disease and serves to protect the host from severe pathology. A significant percentage of people infected with schistosomiasis fail to undergo this protective modulation and can develop portal hypertension with resulting pulmonary complications. Here we show that schistosome-specific antibody-secreting B cells accumulate in the liver as the infection progresses to the chronic state and that this accumulation is dependent on the cytokine Interleukin-10. Blocking the IL-10R results in not only the loss of B cells from the liver, but also the development of severe pulmonary pathology. We found similar changes in disease progression in mice genetically unable to mount normal antibody responses. We believe that antibody is important for triggering the production of anti-inflammatory factors, including IL-10 itself, by other immune cells called macrophages. Our data suggest that during chronic schistosomiasis IL-10 promotes the development of a population of B cells within the liver that is responsible for minimizing inflammation and preventing the development of disease in the lungs. Our findings provide a mouse model that may be of use for studying the development of pulmonary complications due to chronic schistosomiasis.

## Introduction

Schistosomiasis, a chronic neglected tropical disease caused by helminth parasites of the genus *Schistosoma*, affects approximately 200 million people worldwide, is associated with high morbidity, and leads to more than 300,000 deaths per year in Africa alone [1], [2], [3]. Mature *Schistosoma mansoni* worm pairs live in the portal vasculature, producing eggs, which are able to transit from the lumen of the blood vessels to the intestine. Eggs excreted with feces allow transmission of the infection. Since blood flows towards the liver in the portal system, many eggs fail to engage the intestine and instead are carried to the liver where they become trapped in the sinusoids. Egg antigens elicit strongly Th2-polarized cellular responses which orchestrate the development of granulomatous lesions around tissue-trapped eggs [4]. The Th2 response is essential for host survival [5], [6], [7] but also leads to hepatic fibrosis during chronic infection due primarily to the profibrotic effects of IL-13, a major cytokine product of Th2 cells [6], [8], [9]. Granulomatous inflammation is typically modulated as the disease progresses to the chronic state, an effect that is associated with development of hyporesponsiveness within the Th2 cell population [10]. Extensive hepatic fibrosis is associated with hepatosplenic schistosomiasis, a form of the disease that occurs at a frequency of 5 – 10% in untreated infected populations and which has a high mortality rate [11]. Hepatosplenic disease is generally thought to reflect a failure to modulate the immune response over time, with the consequence that immunopathology is particularly severe [12], [13]. Another form of severe schistosomiasis has been recognized, in which patients develop pulmonary hypertension [14], [15], [16]. This condition afflicts up to 20% of individuals with hepatosplenic disease, but is poorly defined and understudied.

There is evidence from both experimental infections in mice, and from studies of infected people, that IL-10 plays a protective immunomodulatory role during schistosomiasis [13], [17], [18]. Here we set out to re-examine whether IL-10 signaling limits severe pathology during chronic schistosomiasis primarily by inhibiting Th2 cell proliferation, a previously proposed mechanism of action [19], [20]. Our data show clearly that specific blockade of IL-10 signaling *in situ* by administration of an anti-IL-10 receptor (IL-10R) mAb to chronically infected mice has no measurable effect on Th2 cell proliferation or IL-4 production (a measure of activation). However, during the course of these experiments we noted that chronic infection is associated with the striking accumulation of schistosome egg antigen (SEA)-specific IgG1-secreting B cells in the liver, and that consistent with a role for IL-10 in plasma cell development [21], [22], this is inhibited by IL-10R blockade. Unexpectedly, IL-10R blockade during chronic infection led to increased morbidity due to the development of severe pulmonary disease associated with the portosystemic shunting of parasite eggs to the heart and lungs. A role for B cells in the prevention of these severe sequelae is indicated by the finding of similar disease in chronically infected B cell-deficient or AID-/- animals. Our data indicate that IL-10 plays a significant role in preventing the development of pulmonary disease during chronic schistosomiasis and that it does so via a mechanism that is unrelated to Th2 cell hyporesponsiveness. Rather, our findings suggest that IL-10 regulates hepatic humoral immunity and that it is this arm of the immune system that is responsible for initiating anti-inflammatory responses, perhaps mediated by the effects of immune complexes on macrophages, that are essential for preventing the development of severe pulmonary disease.

## Results/Discussion

### Th2 cell hypoproliferation during chronic infection is not IL-10 dependent

**Figure 1:**
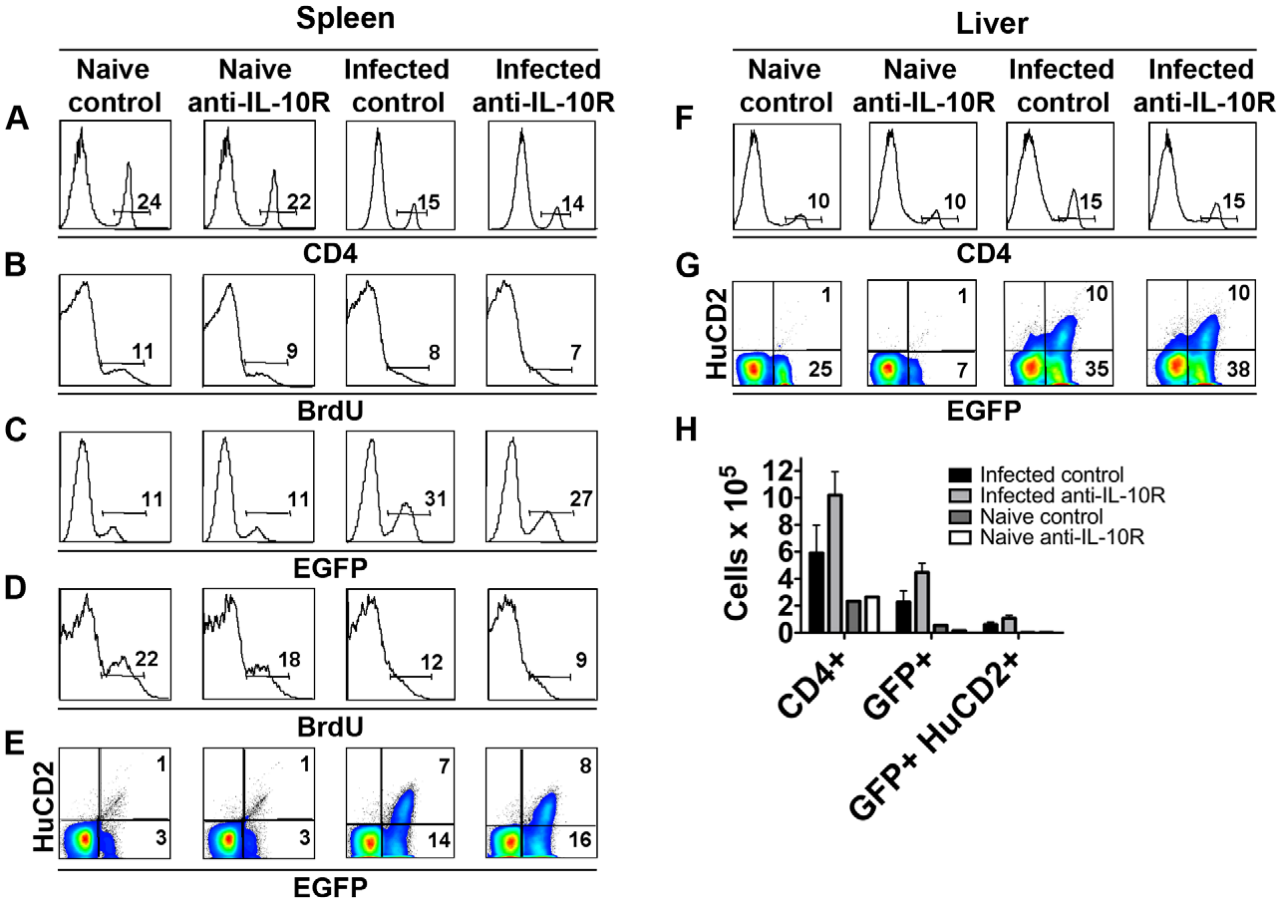
The splenic and hepatic IL-4 response is similar between anti-IL-10R and isotype control treated mice. Balb/c 4get or Balb/c 4get/KN2 mice were infected for 10 – 12 weeks and then treated bi-weekly for 4 weeks with rat anti-IL10R antibody or rat isotype control antibody, and administered BrdU for 7 days before sacrifice. Naïve Balb/c 4get or Balb/c 4get/KN2 mice were similarly treated with antibodies and BrdU. Isolated spleen cells and cells isolated from livers were analyzed by flow cytometry for expression of the markers indicated. Data shown in **A** and **F** are from gated lymphocytes. Data shown in **B** – **E,** and **G** are from gated CD4 T cells. Data shown are concatenated from 3 – 5 mice per group. Numbers show the percentages of cells that fall within indicated gates. **H**, Numbers of CD4^+^, GFP^+^ CD4+ and GFP^+^ HuCD2^+^ CD4+ T cells within liver tissues of infected or naïve mice after treatment with anti-IL-10R or control antibody. Data represent mean plus/minus SD of results from 3 – 5 mice per group. All data shown are representative of three separate experiments with 3 – 5 mice/group.

**Figure 2:**
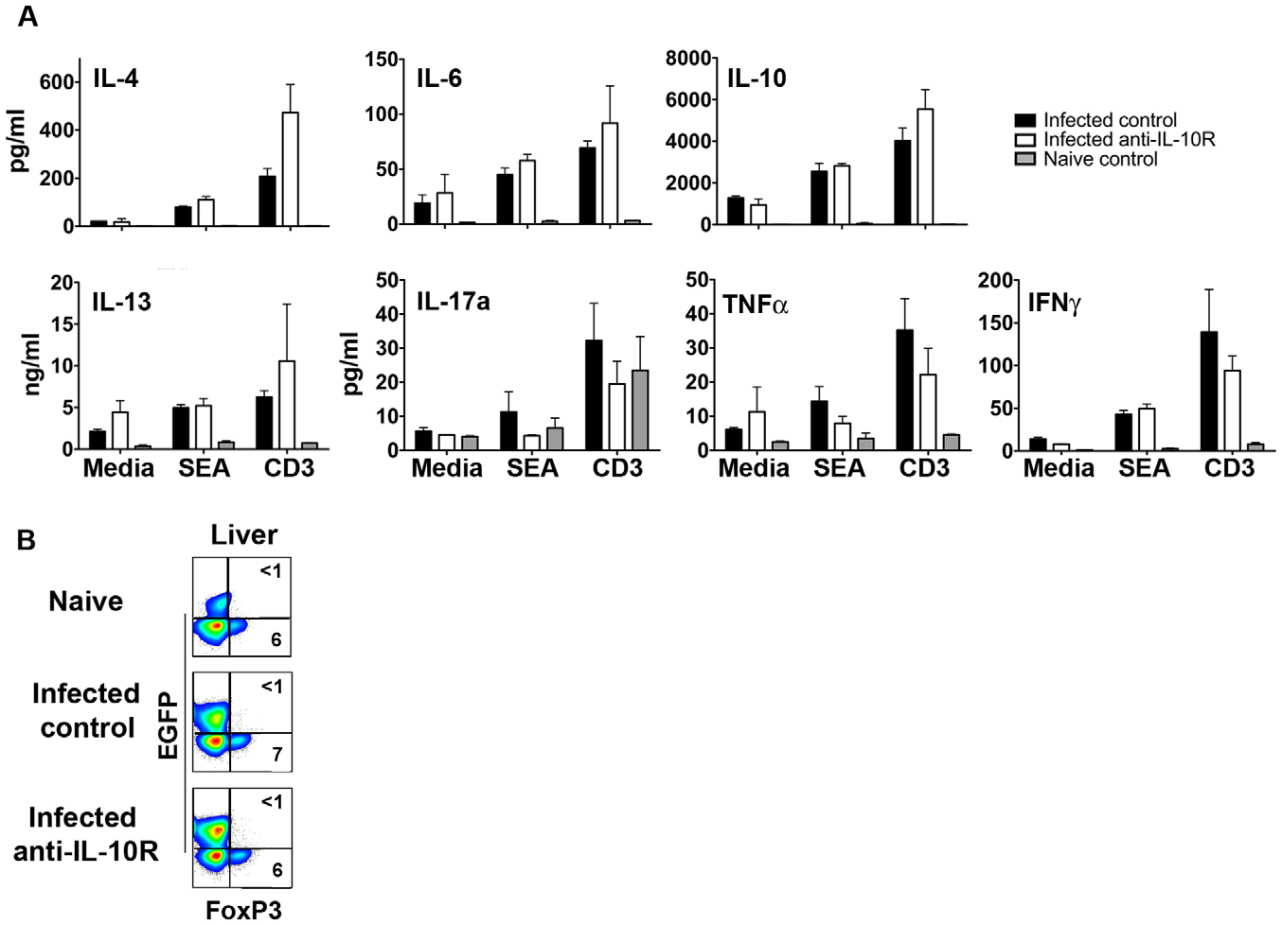
Blockade of IL-10R during chronic infection does not affect hepatic CD4^+^ T cell subset responses. Balb/c 4get/KN2 mice were infected for 11 weeks and then treated bi-weekly for 4 weeks with rat anti-IL10R antibody or rat isotype control antibody. **A,** Purified hepatic CD4^+^ T cells were cultured with TCRα -/- splenocytes and media supplemented with either SEA or anti-CD3 for 72 hours. Cytokine concentrations in culture supernatants were measured. Data represent mean plus/minus SD of results from 4 – 6 mice per group. **B**, Isolated livers cells were analyzed by flow cytometry for expression FoxP3 and GFP. Data shown are from gated CD4^+^ T cells and concatenated from 4 – 7 individual mice per group. Numbers show the percentages of cells that fall within indicated gates.

To assess the effect of IL-10 on Th2 cell responsiveness we infected Balb/c 4get mice (which express EGFP as a reporter for *il4* transcription) or 4get/KN2 mice (which report IL-4 protein production by cell surface expression of human CD2 (HuCD2)) with a low dose of *S. mansoni* and 10 – 12 weeks later initiated a 4 week treatment with blocking IL-10R mAb (1B1.3a [23]). The frequency of CD4^+^ T cells, EGFP^+^ CD4^+^ T cells or of EGFP^+^ huCD2^+^ T cells within the spleens of infected (or naïve) mice was unaffected by IL-10R blockade (Figure 1A, C and E respectively). Consistent with this, the rate of BrdU incorporation by splenic CD4^+^ T cells or EGFP^+^CD4^+^ T cells was also unaltered by treatment with anti-IL-10R mAb (Figure 1B, D respectively); the very low proliferative rate of Th2 cells during chronic infection is apparent in Figure 1D and is consistent with previous reports [10]. We also examined the hepatic CD4^+^ T cell compartment for the effects of IL-10 on T cell function. Blocking IL-10R did not significantly affect the frequency of CD4^+^ T cells in the liver (Figure 1F), the percentages of these cells that expressed EGFP or HuCD2 (Figure 1G), nor the total numbers of Th2 cells (Figure 1H). These data support the view that IL-10 does not enforce Th2 cell hyporesponsiveness during chronic schistosomiasis and are consistent with our recent finding that changes in Th2 cell responsiveness over time during infection are the result of Th2 cell-intrinsic mechanisms such as the expression of GRAIL[10]. We also assessed the effect of blocking IL-10R on the production of Th2 cytokines in addition to IL-4 and of cytokines made by other CD4 T cell lineages (Figure 2A). We found that IL-10R blockade had no effect on the ability of hepatic CD4^+^ T cells to secrete IL-6, IL-10, IL-13, IL-17a, TNFα, or IFNγ in response to restimulation in vitro (Figure 2A). Additionally, IL-10R blockade had no measurable effect on the frequency of FoxP3^+^ regulatory T cells within the hepatic CD4^+^ T-cell compartment (Figure 2B). In summary, measurable effects of L-10R blockade on the hepatic CD4^+^ T cell response during chronic infection were minimal.

### IL-10R blockade during chronic infection induces severe pulmonary and cardiac pathology

**Figure 3:**
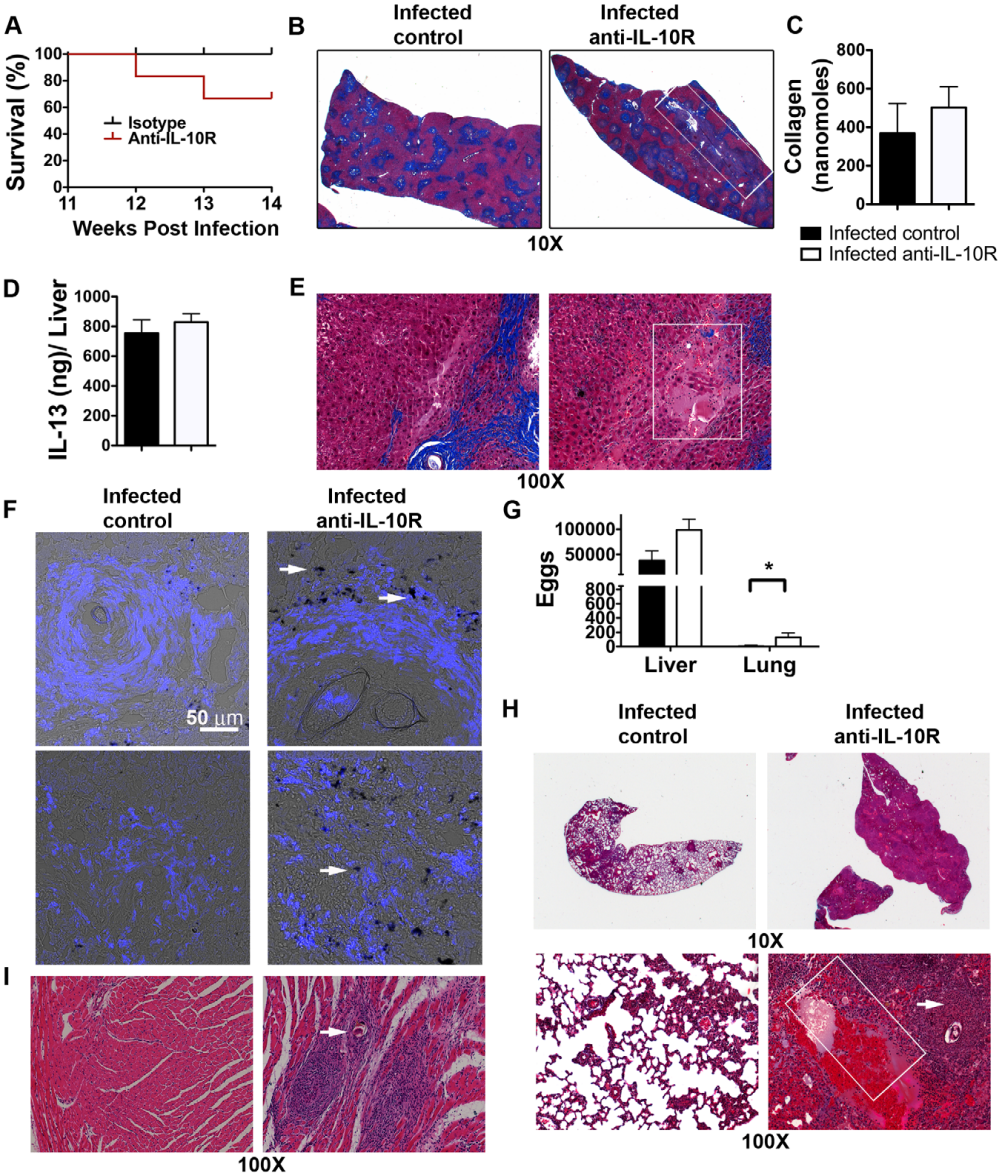
Blockade of IL-10 receptor during chronic infection results in more severe liver disease with pulmonary involvement. Balb/c 4get/KN2mice were injected bi-weekly with anti-IL10R antibody or isotype control antibody during weeks 10 –16 post infection. Naïve Balb/c 4get/KN2 mice were injected with antibodies over the same time period. **A**, Percent survival of infected control or anti-IL0R-treated mice over the course of antibody treatment. Liver sections from control and anti-IL-10R treated animals were stained with Masson's trichrome (**B**, **E**) or hematoxylin and eosin (**H**, **I**). **B**, **E**, Blue staining indicates collagen deposition, white boxes indicate coalescing granulomas (**B**) and hemmorhagic necrosis (**E**). **C**, Hepatic collagen concentrations (nanomoles/liver) in infected control or anti IL-10R treated mice at 16 weeks post infection. **D**, Liver IL-13 concentrations in infected control or anti-IL10R treated mice. **F**, Hemosiderin deposits (arrowheads) in F4/80^+^ cells (blue). **G**, Numbers of eggs in hepatic and pulmonary tissues. Data in **G** represent means of counts from 9 –10 mice per group from two independent experiments, plus minus SD. **H**, Lung sections from infected mice treated with control or anti-IL-10R antibodies. Arrowhead indicates an embolized egg and white box indicates hemorrhage. **I**, Heart sections from infected mice treated with control or anti-IL-10R antibodies; arrowhead indicates parasite egg. Data shown are representative of three separate experiments with 3 – 5 mice/group.

Despite the lack of effect of IL-10R blockade on Th2 cell responsiveness, we observed significant changes in morbidity and mortality associated with the cessation of IL-10 signaling. Specifically, whereas <5% of control-treated mice died before week 14 post infection, >30% of infected mice in which IL-10R was blocked were dead by this time point (Figure 3A); there was no evidence of intestinal hemorrhage in these animals and they did not lose weight precipitously prior to death, which is unlike the situation in, for example, schistosome-infected IL-4^−/−^, IL-4Rα^−/−^ or IL-4^−/−^/IL-10^−/−^ mice, in which death is preceded by severe weight loss [5], [6], [17] (data not shown). Infected mice treated with anti-IL-10R had hepatic granulomas that appeared to coalesce to a greater extent than in infected control mice (Figure 3B), although we were unable to measure increased collagen content, indicative of more severe fibrosis, in these animals (Figure 3C). Moreover, hepatic levels of IL-13, the major inducing factor for fibrosis in schistosomiasis, were also unchanged as a result of IL-10R blockade (Figure 3D). However, we found evidence of hepatic bleeding, apparent as red blood cells and plasma in extravascular spaces with a loss of cellular structure (Figure 3E), and marked deposition of hemosiderin (an indicator of bleeding [24], [25]) (Figure 3F), in mice that were treated with anti-IL-10R. Hemosiderin was localized to F4/80^+^ cells that exhibit macrophage-like morphology (Figure 3F). Notably, IL-10R blockade increased the frequency of F4/80^+^ macrophages at sites distant from granulomas (Figure 3F, bottom). Hepatic bleeding was not due to increased burden of infection since hepatic egg numbers did not differ significantly between anti-IL-10R-treated and infected control mice (Figure 3G). Strikingly, we observed lung inflammation in the infected mice in which IL-10R was blocked that was not apparent in control infected mice (Figure 3H), and digestion of lung tissue revealed significant egg deposition into this organ (Figure 3G). Detailed analysis of the affected lungs revealed eggs as foci of extensive, obliterating cellular infiltration, with areas of hemorrhage; pathologic changes in the lungs of infected mice treated with control immunoglobulin were rare (Figure 3H). Histological examination of the hearts of IL-10R blocked mice revealed eggs and associated cellular infiltrates in 50% of IL-10R blocked animals, whereas inflammation was observed in the heart muscle of only 10% of chronically infected control animals (Figure 3I). The presence of severe pulmonary pathology suggests that these animals may have developed pulmonary hypertension. Pulmonary involvement during schistosomiasis is a result of portosystemic shunting of eggs to the heart and lungs, and reflects increased hepatic-portal blood pressure, which is the typically thought to be the result of liver fibrosis and severe changes to the intrahepatic vascular bed associated with obstructive inflammation [14], [26], [27], [28]. However, our cumulative findings shown in Figure 3 suggest that IL-10 regulated factors that are not directly linked to increased hepatic fibrosis are able to precipitate increases in portal hypertension and portal-systemic shunting of parasite eggs,

Previous reports have shown that compared to infected wild type mice, mice genetically deficient in IL-10 develop larger hepatic granulomas during acute infection and exhibit higher mortality rates during chronic infection [17]. Consistent with these data, the injection of IL-10/Fc fusion protein during acute infection results in decreased granuloma size [29], while the blockade of IL-10R during acute infection results in increased granuloma size [19]. In humans, severe periportal fibrosis during chronic schistosomiasis is associated with lower levels of IL-10 production, and it has been suggested that IL-10 also plays a role in preventing fibrosis in infected mice [20], [29]. In mice, IL-10 has been shown to cooperate with IL-12p40 and IL-13Rα2 to suppress fibrosis due to schistosomiasis [30]. Nevertheless, despite evidence that IL-10 is anti-fibrotic in pulmonary tissues [31] and in liver fibrosis caused by carbon tetrachloride [32], there is no published evidence that mice that lack IL-10 alone develop more extensive fibrosis during the acute or chronic stages of schistosomiasis [17], [33]. Our studies on chronic infection in Balb/c mice, which differ from previous studies in which the effects of IL-10 in chronic infection in B6 mice or acute infection in Balb/c mice have been examined, do implicate IL-10 in limiting portal-sytemic shunting during schistosomiasis. Collectively our data show that in Balb/c mice, IL-10 is not required to maintain the modulated state of Th2 cells during chronic schistosomiasis, but does limit other pathological events that lead to increased portal hypertension and severe sequelae. These results are consistent with previous observations of increased portal hypertension and/or mortality when IL-10 is present at low levels or absent during chronic schistosomiasis [13], [17].

### IL-10R blockade alters the hepatic Bcell compartment

Fibrosis and tissue damage during schistosomiasis is immunologically mediated [8]. Since we were unable to detect changes in the Th2 response associated with IL-10R blockade that might account for the observed changes in disease outcome, we performed further analyses to examine whether other components of the immune response were affected by treatment with anti-IL-10R. In the process of analyzing the hepatic cellular infiltrate, we noticed striking and previously unrecognized differences within the B cell compartment as a result of infection. Moreover, we noted marked effects of IL-10R blockade on the hepatic B cell compartment.

**Figure 4:**
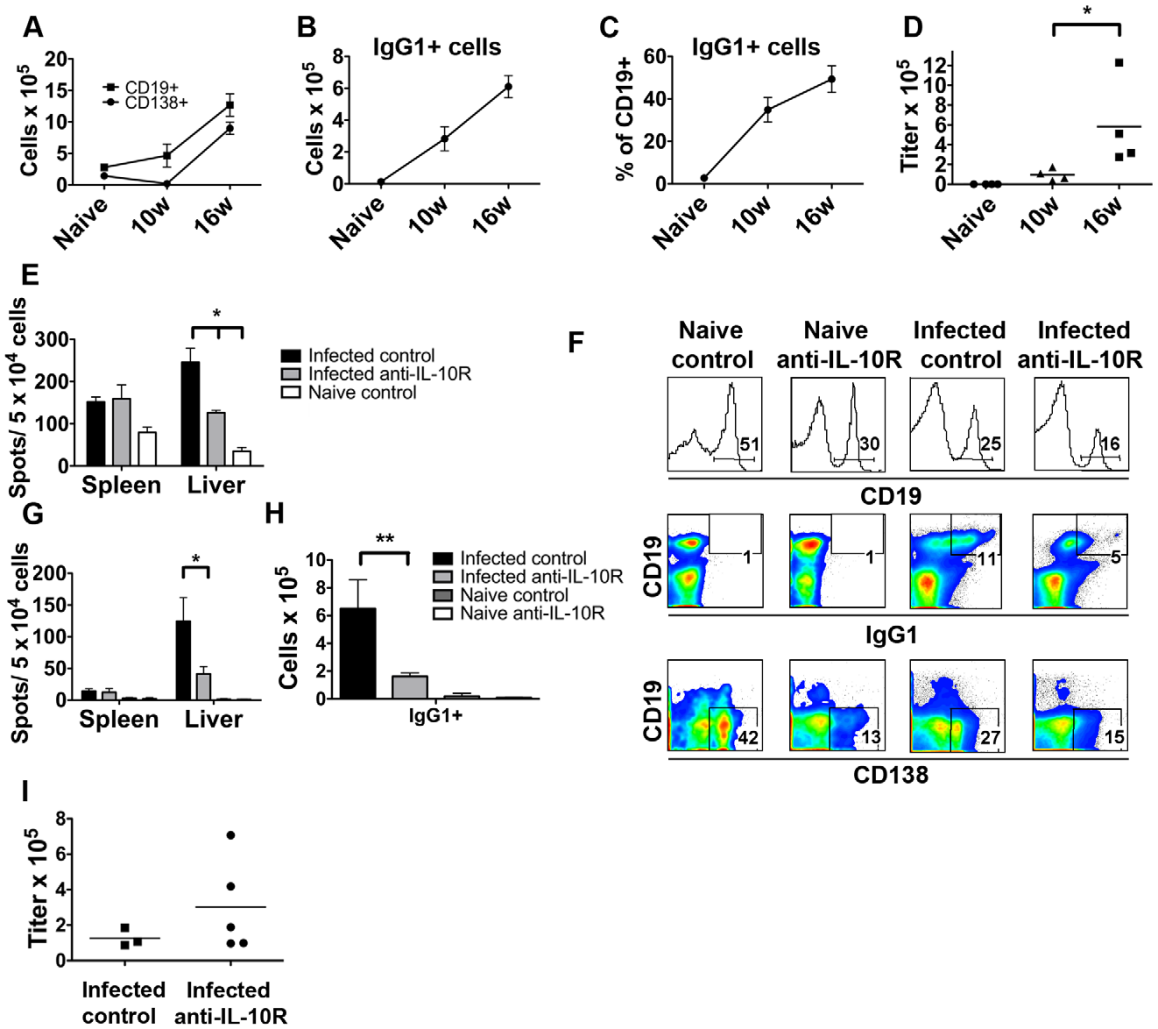
Blocking IL-10R during chronic infection results in alterations within the hepatic B cell compartment and a reduction in the production of IgG1 within the liver. **A**, Numbers of hepatic CD19^+^ B cells and CD138^+^ plasma cells during the course of infection in Balb/c 4get/KN2 mice. **B**, Numbers of surface IgG1^+^ CD19^+^ B cells, and **C**, percentages of CD19^+^ B cells that are surface IgG1^+^ in the liver over the course of infection. Data points represent means plus/minus SD from 4 animals for each time-point. **D**, Serum SEA-specific IgG1 antibody titers over the course of infection. Data shown are from individual mice with mean values represented by horizontal bars. **E**, **G**, Perfused livers from mice treated with anti-IL-10R or control antibody as described in the legend for Fig 3 were digested with collagenase and isolated leukocytes were assayed for total IgG1-secreting cells (**E**) or total SEA-specific IgG1 secreting cells (**G**) by ELISPOT. **F**, Cells isolated from livers were analyzed by flow cytometry for expression of the markers indicated. Data shown are concatenated plots from 3–5 mice and are gated on live lymphocytes defined by FSC/SSC. For analysis of CD138^+^ cells the population was further gated on surface IgD^−^ cells. Numbers show the percentages of cells that fall within the indicated gate. **H**, Numbers of hepatic surface IgG1^+^ B cells at 16 weeks post infection in control and IL-10R blocked animals. Data graphed are the mean plus/minus SD of 3 – 5 mice per group. **I**, Serum SEA-specific IgG1 antibody titers at 16 weeks post infection. Data shown are from individual mice with mean values represented by horizontal bars. All experiments were performed at least 3 times. * indicates P<0.05, ** indicates P<0.01.

Consistent with previous reports [34], [35], we found that infection induced the accumulation of CD19^+^ B cells within the liver (Figure 4A). Interestingly, much of this expansion occurred late in infection between weeks 10 and 16 (Figure 4A), lagging behind the peak of granulomatous inflammation which occurs at week 8 of infection (not shown; [4]). The presence of plasma cells within the liver, defined by CD138 staining, followed a similar pattern (Figure 4A). Th2 cells dominate the T cell response during schistosomiasis [4], and the Th2 cell product IL-4 promotes class switching to IgG1 [36]. Consistent with this, numbers of IgG1+ B cells in the liver increased significantly as a result of infection (Figure 4B) with the result that over 50% of the hepatic CD19^+^ B cells in 16 week-infected mice were IgG1^+^, whereas prior to infection very few of the CD19^+^ cells expressed this isotype (Figure 4C). Despite the fact that the overall numbers of B cells in the liver did not increase dramatically by week 10 of infection (Figure 4A), there was a transition within this population in terms of IgG1^+^ cells such that approximately 37% of CD19^+^ cells at week 10 were IgG1+ compared to <2% in naïve mice (Figure 4C). Consistent with the hepatic plasma cell data and previous reports [37], schistosome egg antigen (SEA)-specific serum antibody titers were also found to increase strikingly between weeks 10 and 16 of infection (Figure 4D). Targeted measurements of antibody-secreting cells by ELISpot revealed large numbers of IgG1-secreting cells from the perfused livers of infected mice that, on a per cell basis, exceeded the number of IgG1-secreting cells from the spleens of infected animals (Figure 4E). Moreover, while only a small percentage (10%) of IgG1-secreting cells in the spleen were making antibody that recognized SEA, >50% of IgG1-secreting hepatic B cells produced SEA-specific antibodies (Figure 4G, and see Figure 4E). The hepatic pool of antibody secreting cells is therefore highly enriched for those secreting antibody specific for the egg stage of the parasite. These data emphasize previously unappreciated changes in the liver B cell compartment during infection that result in the accumulation of pathogen-specific IgG1-secreting plasma cells. This is consistent with reports that plasma cells can be recruited to tissue sites of inflammation [38], [39].

IL-10R blockade had a marked effect on hepatic B cells. The frequencies of CD19^+^ B cells and CD138^+^CD19^−^ plasma cells within hepatic leukocyte populations from naive and/or chronically infected mice were significantly reduced as a result of treatment with anti-IL-10R (Figure 4F). Similarly, the overall numbers of hepatic CD19^+^IgG1^+^ B cells (Figure 4H), and of IgG1-secreting cells (Figure 4E) in chronically infected mice were decreased by IL-10R blockade. Further, anti-IL-10R treatment caused a three-fold reduction in the frequency of SEA-specific IgG1-secreting cells in the liver (Figure 4G). Similar results were obtained upon examination of hepatic IgE and IgM-secreting cells from IL-10R-treated mice (Figure S1). Interestingly, there was no effect of blocking IL-10R on splenic IgG1, IgE, or IgM-secreting cell numbers (Figure 4E, G, Figure S1), and overall peripheral serum anti-SEA IgG1 antibody titers did not decline following anti-IL-10R treatment (Figure 4I).

There are several explanations for the impact of IL-10R blockade on the hepatic B cell compartment. The first is that IL-10R signaling is required for the recruitment of plasma cells to the liver, but not for their generation in the spleen or lymph nodes. Previous reports have shown that IgG-secreting cells generated at non-mucosal surfaces express α_4_β_1_ which allows them to home to bone marrow and other tissues through interactions with VCAM1, expression in the liver of which is increased during schistosomiasis [40], [41]. Alternatively, hepatic plasma cells might originate from class switched circulating memory B cells or recent germinal center emigrants that are recruited to the liver, and differentiate into plasma cells upon antigen re-stimulation [42], [43], in an IL-10R dependent manner. This possibility seems likely as we see a dramatic reduction in the number of IgG1^+^ B cells (Figure 4 H) in the liver after blocking IL-10R during chronic infection. Previous reports have documented a role for IL-10 in plasma B cell development [21], [22]. Moreover, differentiation of B cells into IgA-secreting plasma cells within the gut lamina propria has been reported [44] making it formally possible that *S. mansoni*-induced hepatic IgG1 antibody secreting cells are generated in the liver from B cells first activated in lymphoid organs. Alternatively, IL-10 may be regulating the production of chemokines that are essential for B cell entry into the liver. One candidate for this would be CXCL13 [45], production of which is induced in the hepatic tissues of mice infected with *S. japonicum*, which is closely related to *S. mansoni* [41], [46].

### Chronic infection leads to the hepatic accumulation of IgG1+F4/80+ cells

**Figure 5:**
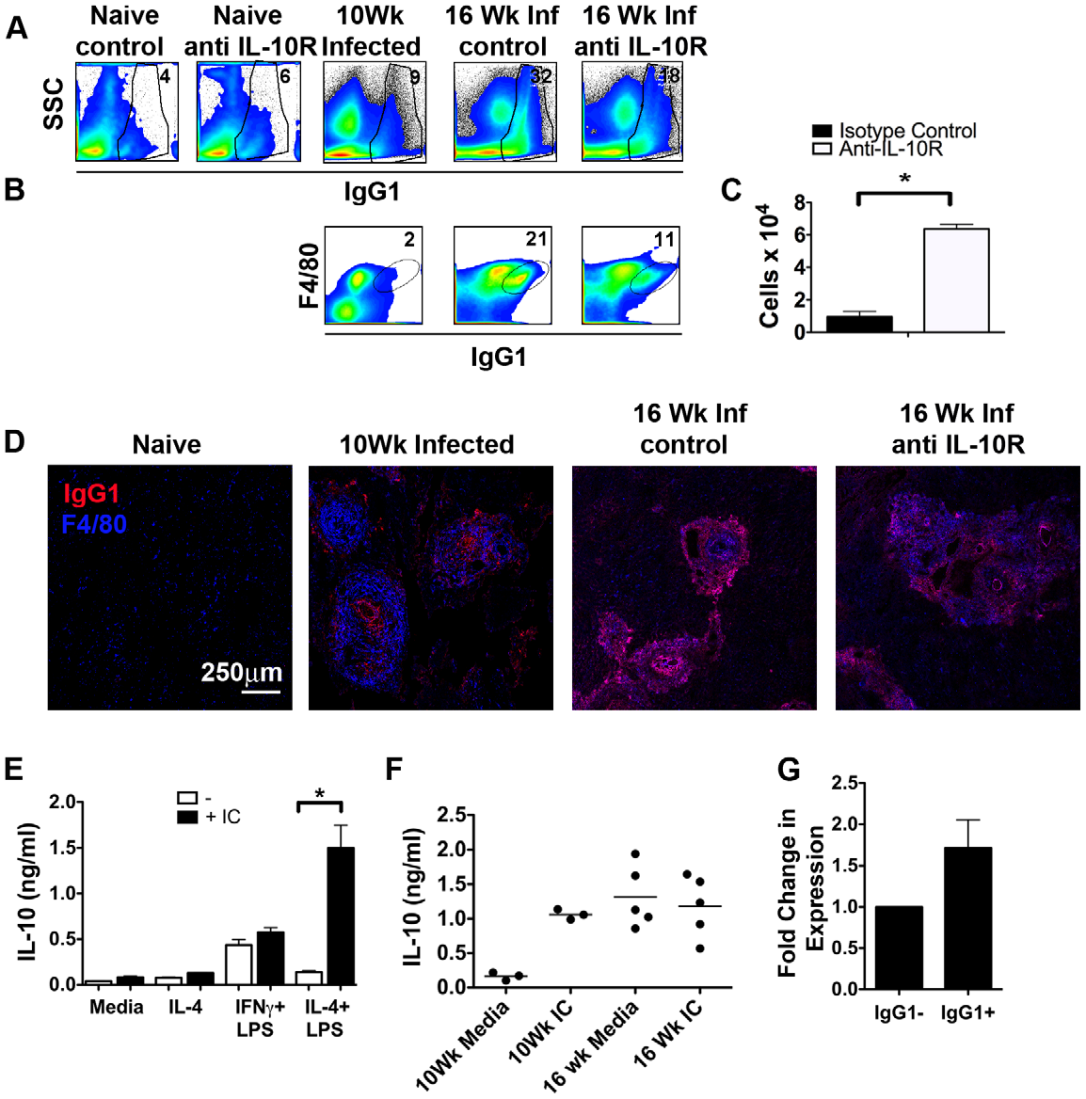
IgG1 localizes to F4/80+ cells in the liver during chronic infection and IgG1 binding up-regulates IL-10 production. **A**, Cells isolated from livers of naïve or infected Balb/c 4get/KN2 animals treated with anti-IL-10R antibody or with control antibody were stained for surface IgG1 and analyzed by flow cytometry. **B**, Frequency of IgG1^+^ F4/80^+^ cells during S. mansoni infection. The F4/80 positive cells are also Ly6c, CD11b, and MHCII positive (not show). Data shown are concatenated plots from 3 – 5 mice, and are gated on live cells. Numbers show the percentages of cells that fall within each gate. **C**, Numbers of hepatic CD11c^+^F4/80^Hi^ macrophages (gated on Ly6c^Hi^ CD11b^Hi^) after treatment with anti-IL-10R or control antibody. **D**, Frozen liver sections were stained for IgG1 (Red) and F4/80 (Blue) and imaged with a Leica TCS SP5 II laser scanning confocal microscope at the indicated times post *S. mansoni* infection. Cells that are both IgG1^+^ and F4/80^+^ appear as magenta. Data shown are representative of two experiments with 3–5 animals per group. **E**, Balb/c bone marrow derived macrophages were primed in the indicated conditions and then stimulated for 20 hours with either SEA immune complexes (IC) or media and the supernatants assayed for IL-10 production. **F**, Liver cell suspensions from *S. mansoni* infected livers (either 10 or 16 weeks post infection) were depleted of B and T cells and then cultured for 20 hours with either media or SEA immune complexes and the supernatants assayed for IL-10 production. **G**, Liver cell suspensions for 16 week infected Balb/c mice were stained for Ly6c, CD11c, CD11b, F4/80, and IgG1, and then IgG1^+^ and IgG1^−^ macrophages (Ly6c^Hi^/CD11b^Hi^/CD11c^+^F480^+^) were sorted for RNA extraction. Expression of IL-10 is normalized to the housekeeping gene β-actin. Data shown for G are combined from two individual sorts of 5–7 animals.

When analyzing IgG1-expressing cells during infection we noted that IL-10R blockade affected a large population of liver-infiltrating IgG1^+^ non-B cells (CD19^−^, CD138). This population was heterogeneous, as indicated by side scatter (Figure 5A), but largely F4/80^+^ Ly6c^Hi^, CD11b^+^, MHCII^HI^, and increased over time (Figure 5B and data not shown). Consistent with the observed effects of IL-10R blockade on the hepatic IgG1-secreting B cell population, the frequencies of IgG1^+^ non-B cells, and of F4/80^+^IgG1^+^ cells in particular, were significantly decreased as a result of the treatment of infected mice with anti-IL-10R (Figure 5A, B). Conversely, the total number of CD11c^+^F4/80^Hi^ macrophages were significantly increased in IL-10R blocked animals (Figure 5C), a finding that supports the qualitative differences in the frequency of F4/80^+^ cells indicated by Figure 3C. F4/80^+^IgG1^+^ cells were clearly evident in sections of livers from mice infected for 16 weeks and were localized primarily to granulomatous lesions (Figure 5D). Consistent with our flow cytometric data (Figure 5A,B), IgG1^+^ cells were rare in sections of livers from mice infected for 10 weeks, and tended to be located immediately around parasite eggs and to not be F4/80 positive, despite the fact that the granulomas at this time point were also macrophage-rich (Figure 5D). Again consistent with the flow cytometric data, F4/80^+^IgG1^+^ cells were observed far less frequently in chronically infected mice in which IL-10R was blocked, than in control infected mice (Figure 5D). Staining of livers from naïve mice show a small population of F4/80^+^ resident macrophages, but no IgG1^+^ cells (Figure 5 D). This control staining combined with analysis of liver sections from infected B cell deficient µMT mice (data not shown) indicate that the observed IgG1 staining and IgG1 and F4/80 co-localization was specific.

Overall, data from the flow cytometric and immunohistochemical analyses support the conclusion that as infection progresses from week 10 to week 16, there is an IL-10-dependent increase in IgG1-producing B cells within diseased liver tissue, and that IgG1 made locally becomes associated with macrophages. It has previously been reported that regulatory macrophages can be generated by immune complex stimulation, and that these macrophages are able to then produce IL-10 [47], [48], [49]. We reasoned that IgG1^+^ macrophages in the livers of infected mice could be contributing to the regulation of inflammation by secreting IL-10. To begin to assess whether this pathway is active during chronic *S. mansoni* infection, we generated bone marrow derived macrophages and assessed their ability to produce IL-10 in response to SEA-containing immune complexes following priming by IFNγ and LPS (classical activation), IL-4 (alternative action) or exposure to IL-4 and LPS, which mimics the situation in vivo during infection [7], [50]. We found that macrophages stimulated with IL-4 and LPS responded strongly to immune complexes by making IL-10 (Figure 5E). We then assed the ability of macrophage-enriched single cell suspensions from the livers of infected mice to secrete IL-10 in response to immune complexes. Cells from mice infected for 10 weeks, which would not be expected to have acquired immune complexes *in vivo* (Figure 5A,B,C), made little IL-10 *ex vivo* but responded strongly to added immune complexes by secreting this cytokine. In contrast, cells from mice infected for 16 weeks, which are IgG1 positive *in vivo*, made IL-10 *ex vivo* without exogenous stimulation, and no enhanced effect was observed with the addition of immune complexes (Figure 5F). These data indicated that IgG^+^ macrophages are likely to be making IL-10 *in situ* in chronically infected mice. To examine this, we used real time RT-PCR to measure IL-10 transcripts in IgG1^+^ vs. IgG1^−^ macrophages isolated from diseased liver tissues from chronically infected mice. We found that IL-10 mRNA expression was higher in sorted liver IgG1^+^ (Ly6c^Hi^ CD11b^Hi^ CD11c^+^ F4/80^+^) macrophages from 16 week infected Balb/c mice compared to IgG1^−^ (Ly6c^Hi^ CD11b^Hi^ CD11c^+^ F4/80^+^) cells from the same mice. (Figure 5G). Taken together, our findings indicate that loss of cells from within the B cell compartment and subsequent reductions in the production and localization of IgG1 within the liver, are striking immunologic correlates of severe disease manifestations following IL-10R blockade. Our data suggest that this localized production of IgG1 generates immune complexes that bind to macrophages and upregulate IL-10 production. Our findings implicate this process in the regulation of immunopathologic changes occurring within the liver during infection, and raise the possibility of the existence of a positive feedback regulatory pathway in which IL-10 promotes local IgG1 production which inturn promotes ongoing IL-10 production by macrophages (Figure S2).

### B-cell deficient mice develop severe pulmonary pathology during chronic infection

**Figure 6:**
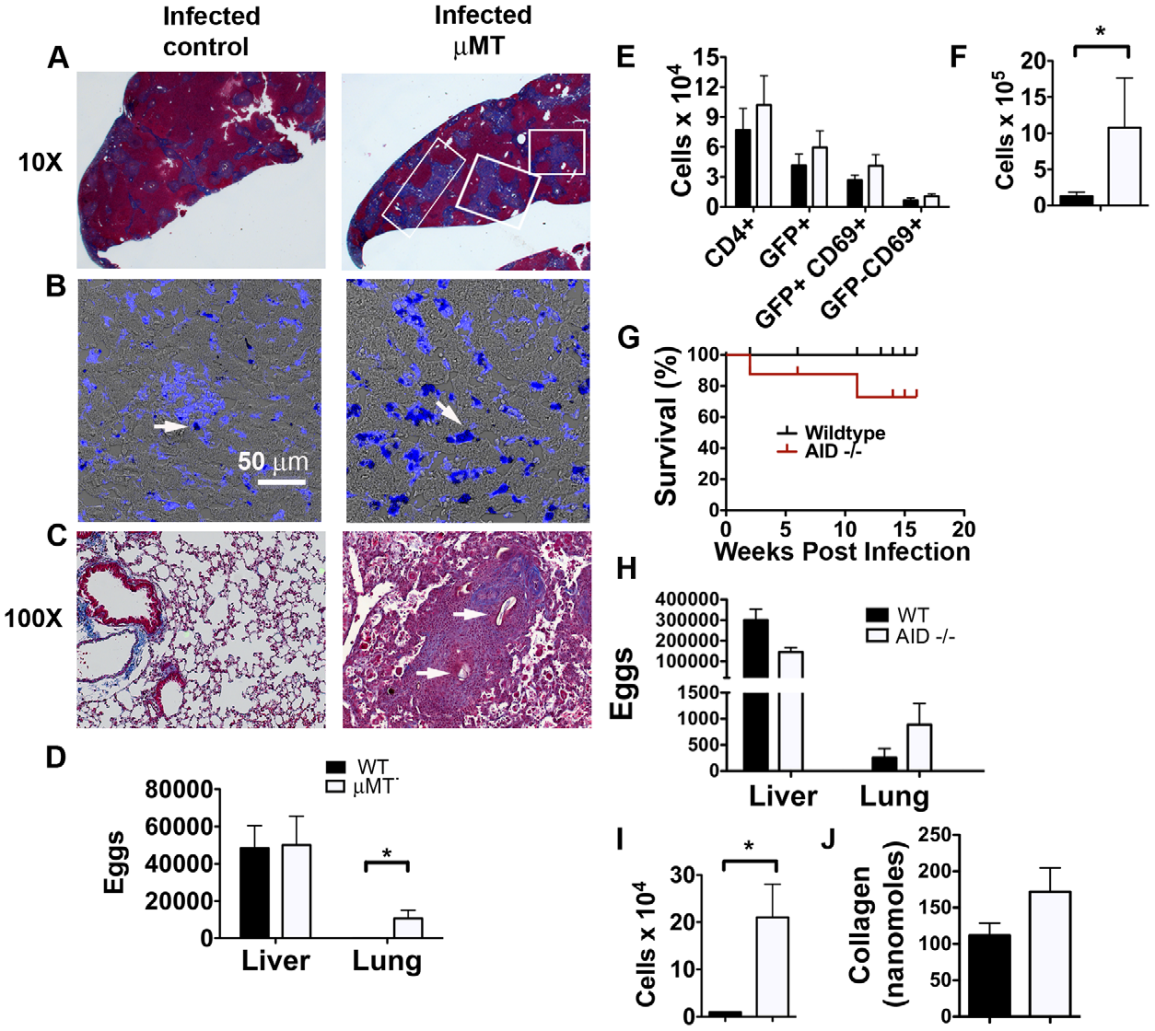
Mice lacking B cells exhibit severe pulmonary pathology during chronic infection. C57BL/6 4get/µMT and C57BL/6 4get controls were sacrificed at 16 weeks post infection. **A**, **C**, Liver and lung sections from control and µMT animals were stained with Masson's trichrome. White boxes indicate coalescing granulomas (**A**) and arrowheads indicate eggs embolized in the lungs (**B**), **C**, Liver sections were stained with an antibody against mouse F4/80 (Blue) and imaged with a confocal microscope. The images shown are overlays of the Blue (F4/80)/DIC channels. Hemosiderin (indicated by arrowheads) crystals are visible under DIC conditions. **D**, Quantitation of hepatic and pulmonary egg deposition in infected control or µMT animals. Data shown are the means plus/minus SD of 9 – 10 mice per group from two independent experiments. **E**, Expression of EGFP, CD4 and CD69 by cells isolated from the livers of infected control and µMT animals were analyzed by flow cytometry. The mean and SD of data from 4 – 5 individual animals per group are graphed. **F**, Numbers of hepatic CD11c^+^F4/80^Hi^ macrophages (gated on Ly6c^Hi^ CD11b^Hi^ cells) at 16 weeks post infection in µMT or control mice. Data are representative of 2 – 3 individual experiments. **G**, Percent survival of infected wildtype control or AID-/- mice following a low dose infection of *S. mansoni*. **H**, Quantitation of hepatic and pulmonary egg deposition in infected control or AID -/- animals. **I,** Numbers of hepatic CD11c^+^F4/80^Hi^ macrophages (gated on Ly6c^Hi^ CD11b^Hi^ cells) at 16 weeks post infection in AID -/- or control mice. Data are representative of 2 individual experiments. **J,** Concentration of collagen (nanomoles/liver) in the livers of control or AID-/- mice at 16 weeks post infection. * indicates P<0.05.

Based on these findings above, we hypothesized that B cells play an important role in preventing the development of pulmonary complications during schistosomiasis. To test this hypothesis, we infected 4get/µMT and wild-type 4get control mice with a low dose of *S. mansoni* and analyzed pathological changes due to chronic infection. As previously published [51], µMT mice were more susceptible to infection than B cell replete mice (20% of infected µMT mice died before week 16 vs. none of the control animals). Increased mortality was associated with the development of tracts of coalescing hepatic fibrosis (Figure 6A), and evidence of increased hemosiderin deposits in F4/80+ macrophages (Figure 6B) indicative of hepatic bleeding, (e.g. [52], [53]). Most significantly, we found that chronically infected µMT mice, like infected wild type mice in which IL-10R was blocked (Figure 3), had eggs in their lungs (Figure 6C, D) with extensive associated cellular infiltration and immunopathologic changes (Figure 6C). In two independent experiments, 89% of µMT animals (n = 9) had eggs in their lungs at 16 – 18 weeks post infection compared to 29% of control animals. All µMT mice that died early during infection (between weeks 11 and 12; 25% of infected µMT mice) had eggs in their lungs and had no indication of intestinal hemorrhage upon necropsy. This was not due to increased hepatic egg burden in the absence of B cells (Figure 6D), nor to a more aggressive Th2 response, since the frequencies and total numbers of EGFP^+^ CD4^+^ T cells, or numbers of CD69^+^ CD4^+^ T cells in the livers of infected µMT mice were no different than in infected B cell-replete animals (Figure 6E). However, as in mice in which IL-10R was blocked, we did note that compared to chronically infected wild type mice, the livers of infected µMT mice were infiltrated by significantly more CD11c^+^F4/80^Hi^ macrophages (Figure 6F).

The lack of significant alterations to the hepatic T cell compartment in infected anti-IL-10R-treated mice and in infected µMT mice, suggests that the increased severity of disease with pulmonary involvement is not due to an increase in immunopathologic hepatic Th2 cell responsiveness. Rather, the changes in the B cell compartment in the infected anti-IL-10R-treated mice and the similarity of the overall disease picture in anti-IL-10R treated and µMT mice indicate that antibody is playing a central role in preventing the development of severe disease. Previous work from our laboratory has shown that CD40L^−/−^ mice fail to produce systemic IgG1 or IgE in response to *S. mansoni* infection, exhibit increased mortality, and strikingly develop egg-associated granulomas in their lungs [54]. The similarity in mortality and morbidity between these mice that do not make IgG1 or IgE during infection, but which do have B cells, and the infected B cell deficient and anti-IL-10R-treated B-cell sufficient mice described herein, suggest that secreted immunoglobulin is involved in preventing the development of pulmonary disease during schistosomiasis. These conclusions are generally consistent with previous reports which have implicated B cells in immunomodulation during chronic schistosomiasis [51], [55], [56]. To directly asses the role of secreted IgG1 in protection against the development of lung pathology during chronic schistosomaisis, we examined the outcome of infection in AID-/- mice, which are unable to undergo class switch recombination or somatic hypermutation [57], and secrete only IgM. AID-/- mice were less capable than wild type mice of surviving into chronic infection (Figure 6G) and were found to have extensive pulmonary disease due to egg deposition into the lungs (Figure 6H and not shown). Compared to wildtype mice, AID-/- mice were similar to infected anti-IL-10R treated and µMT mice in having increased numbers of liver macrophages following infection(Figure 6I). We were unable to correlate increased portal-systemic shunting of eggs with increased liver fibrosis in the absence of IgG1 (Figure 6J and data not shown),

Our data suggest that there are at least two distinct sources and roles for IL-10 during *S. mansoni* infection (Figure S2). The first role is in the development and/or recruitment of class switched hepatic B cells, the numbers of which are reduced in the liver after IL-10R blockade, in the absence of any changes in the broader systemic of B cell pool. The source of the IL-10 that is important for this process is currently unclear. However, Th2 cells make IL-10 and are present during infection from early time points following the onset of egg production, so it is reasonable to hypothesize that Th2 cells are responsible for the IL-10 that is important for the hepatic B cell response. Our time course experiments indicate that class switched B cells appear in the liver before plasma cells do, and that hepatic F4/80^+^IgG1^+^ macrophages are difficult to detect until after a population of plasma cells has accumulated. Since systemic egg antigen-specific IgG1 is measurable prior to the appearance of plasma cells in the liver, it seems likely that IgG1 is not present within this tissue until antibody-secreting plasma cells localize to the organ. IgG1^+^ macrophages are an additional source of IL-10, but whether these cells or the IL-10 made by them plays a direct role in preventing the development of portal hypertension remains to be determined. However, it has been reported that *S. mansoni*-infected FcRγ^−/−^ mice develop similar disease to that evident in infected µMT mice [51], supporting the view that an FcγR^+^ cell type is mediating the beneficial effects of antibody. Several types of myeloid cells, including regulatory macrophages [58], [59], [60] and/or myeloid-derived suppressor cells [61], are candidates for cells that express FcγRs and either make or are dependent on IL-10, and that may play a protective role in the chronically inflamed tissues of schistosome-infected mice. Macrophages have been proposed as the “master regulators” of fibrosis secreting both pro- and anti-fibrotic mediators [62]. Recent work has revealed that alternatively activated macrophage-derived Arginase-1 (Arg-1) is involved in the down-regulation of granulomatous inflammation and fibrosis, and that the macrophage specific deletion of Arg-1 leads to increased mortality and hepatic fibrosis/necrosis [63]. Other work suggests that there is functional plasticity in macrophages such that when IL-4 primed (alternatively activated) macrophages are cultured with immune complexes they take on a “hybrid” phenotype where they produce IL-10, but still express some markers of alternative activation [64], [65]. Our finding that reductions in the numbers of IgG1^+^ macrophages are associated with the development of severe pathology suggests that during chronic *S. mansoni* infection, alternatively activated macrophages may bind immune complexes via FcγRs and assume regulatory/anti-inflammatory roles. This possibility is bolstered by our finding that immune complexes upregulate IL-10 secretion when added to liver cells from acutely infected mice, and that sorted IgG1^+^ macrophages from 16 week infected mice express more IL-10 mRNA than IgG1^−^ macrophages. In the absence of IgG1, there is a significant increase in the number of CD11c^+^F4/80^hi^ cells within diseased liver tissues, suggesting that immune complex ligation modulates either the recruitment or proliferation of these cells during chronic infection. We speculate that in the absence of immune complex-initiated signaling events, macrophages that would normally serve an anti-inflammatory role either lose this ability or become proinflammatory. Current work is directed towards more carefully defining the macrophage subpopulations that our findings here implicate in the regulation of disease progression during schistosomiasis mansoni, and elucidating the roles of IL-10 in the orchestration of the hepatic B cell compartment.

## Materials and Methods

### Ethics statement

This study was carried out in strict accordance with the recommendations in the Guide for the Care and Use of Laboratory Animals of the National Institutes of Health. The protocol was approved by the Institutional Animal Care and Use Committee of Trudeau Institute (Permit Number: 09-007). All efforts were made to minimize suffering.

### Mice and parasites

4get (C.129- *Il4tm1Lky*/J) and 4get/KN2 mice were previously described [66], [67]. 4get/µMT and AID-/- [57] mice on a B6 background, and Balb/c TCRα -/- mice, were bred at Trudeau Institute. All experimental procedures with mice were approved by the Institutional Animal Care and Use Committee of the Trudeau Institute. Mice were kept under specific pathogen–free conditions and were infected at 8–12 weeks of age. Mice on a Balb/c background were exposed percutaneously to 35 *Schistosoma mansoni* (Puerto Rican strain, NMRI) cercariae and mice on a B6 background were exposed to 50 cercariae. For IL-10R blocking, animals were given bi-weekly i.p. injections of either 250 µg of purified anti-IL-10R monoclonal antibody (clone 1B1.3A) or 250 µg of purified rat IgG1 isotype control antibody. Soluble egg Ag (SEA) was prepared from isolated schistosome eggs as previously described [10], [54].

### Histology and egg counts

Liver, lungs and heart were collected from PBS-perfused animals and immediately fixed in 10% neutral buffered formalin. Tissues were embedded and sectioned, and sections were stained with hematoxylin and eosin or Masson's trichrome for enumeration of inflammatory infiltrate and collagen deposition. The extent of liver fibrosis in anti-IL-10R or isotype control treated animals at 16 weeks post-infection was determined by measuring the percentage of fibrotic tissue in trichrome stained liver sections [68]. Samples of lung and liver were collected to quantitate egg deposition as previously described [69].

### In vivo BrdU labeling

Mice were injected i.p. with 0.5 mg BrdU (BD Biosciences or Sigma-Aldrich) at the start of the labeling period and thereafter provided with 0.8 mg/ml BrdU in their drinking water. Fresh water with BrdU was provided every 2–3 days.

### Flow cytometric analysis

To analyze hepatic cell populations livers were removed from PBS-perfused animals, mashed, and incubated in RPMI (Mediatech) containing 250 µg/ml Collagenase D (Roche) at 37°C for 60 min. The resulting suspension was disrupted through a 100 µm metal cell strainer and centrifuged through 40% isotonic Percoll/RPMI. The resulting pellet was washed, and used for analyses. Spleen cells were harvested as previously described [10]. Surface staining with monoclonal antibodies, acquisition, and analyses were essentially performed as previously described [10]. Samples were acquired using a FACSCanto II flow cytometer (BD) and analyzed with FlowJo software (Tree Star, Inc.). The following mAb (BD, eBioscience, BioLegend, or Invitrogen) against mouse antigens were used as PE, PE-Cy5, PE-Cy7, allophycocyanin (APC), APC-Cy7, Pacific blue, or biotin conjugates: CD4 (RM4-5), CD19 (1D3), CD138 (281-2), IgG1 (A85-1), IgD (11-26), IgM (11/41), CD11b (M1/70), CD11c (N418), F4/80 (BM8), IA/IE (MHCII; M5/114.15.2), Ly6c (HK1.4), BrdU (MoBu-1) FoxP3 (FJK-16s), and huCD2 (RPA-2.10). Biotinylated antibodies were secondarily stained with APC-Cy7-conjugated streptavidin. Plots shown are on a Logicle scale.

### Immunohistochemistry

Small pieces (0.5 cm^2^) of PBS-perfused livers were immediately frozen in optimal cutting temperature (OCT) embedding compound (Sakura Finetek) over liquid nitrogen. Frozen livers were cut into 7 µm sections on a Leica cryostat. Sections were fixed in a mixture of ice-cold 75% acetone/25% ethanol for 5 min. Sections were blocked with PBS containing 2% normal mouse serum and 2% normal donkey serum overnight at 4°C. Sections were stained with rat anti-mouse IgG1 (A85-1, BD) and rat anti-mouse F4/80 (BM8, Biolegend) in blocking buffer for 60 min, washed with PBS. Images were generated with Leica LAS AF 2.1.1 software, using the tile scan feature to stitch together 25 images (5x5) taken with a 20x objective 0.7NA at a resolution of 1024×1024.

### ELISA and ELISPOT

SEA-specific serum IgG1 endpoint titers were determined by ELISA using the IgG1-specific mAb X56 (BD); Immulon 4HBX plates (Thermo Fisher Scientific) were coated overnight at 4°C with 0.2 µg of SEA/well, blocked with FBS, and incubated with serial dilutions of sera, followed by a peroxidase coupled anti-mouse IgG1 and ABTS substrate. Total protein was extracted from perfused livers in Tissue Extraction Reagent I (Invitrogen), per manufacturer instructions. Liver extract and culture supernatant IL-13 concentrations were determined using the IL-13 duo set (R&D) as per manufacturer's instructions. IL-10 concentration in culture supenatants was measured using capture and detection antibodies from R&D as per manufacturer instructions. For ELISPOTs, single-cell suspensions of splenocytes or collagenase digested liver cells from infected Isotype control or IL-10R blocked mice were cultured in RPMI 1640 supplemented with FCS for 24 h in MultiScreen-HA plates (Millipore, Billerica, MA) coated with 2 µg/ml of either anti-mouse IgG1 (BD Biosciences), anti-mouse IgE, anti-mouse IgM, or SEA. Bound Abs were detected with non-competing HRP labeled anti-mouse IgG1, IgM, or IgE (SouthernBiotech). Bound antibody was detected with the AEC Chromogen Kit (Sigma) per manufacturer instructions and spots were counted using Immunospot analyzer (v4.1, C.T.L, Cellular Technology Limited).

### Macrophage cultures

Macrophages were differentiated from bone marrow in a 6-day protocol as previously described [47], and stimulated with either IFNγ followed by LPS (to generated classically activated macrophages), or IL-4 with or without LPS stimulation (to generate alternatively activated macrophages) as previously described [47]. Following this stimulation, 5 µl of immune complexes (generated by mixing 50 µg of SEA with 20 µl of 16 week infected serum at room temperature for 30 min [47]) were added to the experimental wells for 24 hours, after which culture supernatants were assayed for IL-10. For liver cell experiments, B and T cells were depleted from digested liver cell preparations using Dynabead mouse pan B and pan T depletion kits (Invitrogen) as per manufacturer's instruction. The resulting cells were cultured for 20 hours (90,000 cells per well) with complete macrophage media [47] with our without immune complexes (generated as above), after which the culture supernatants were assayed for IL-10.

### CD4 T-cell restimulation

CD4^+^ T cells were sorted from livers of naive, infected control or IL-10R blocked animals using a BD Influx. Sorted cells were >95% pure. Purified CD4^+^ cells were cultured at a 10∶1 ratio with splenocytes from Balb/c TCRα -/- mice and stimulated with either SEA, α-CD3 or media alone for 72 hours. The supernatants were harvested and concentrations of IL-4, -6, -10, -17a, TNFα and IFNγ were measured using the BD Mouse TH1/TH2/TH17 cytokine bead array kit as per manufacturer's instructions, and IL-13 was measured as above.

### RNA isolation and purification and RT PCR

Macrophages were sorted from 16 week infected Balb/c mice based on surface expression of Ly6c, CD11c, CD11b, F4/80, and IgG1 using a BD Influx. Sorted cells were >95% pure. RNA was isolated from sorted cells resuspended in 0.5mL Trizol (Invitrogen) using manufacturer's instructions and Qiagen's RNeasy Micro “RNA clean-up” protocol with an on-column DNase treatment. First strand cDNA was synthesized using isolated RNA, Superscript II reverse transcriptase (Invitrogen), and oligo dT as a primer. Taqman assays were performed using Applied Biosystems' 7500 real-time PCR system and Taqman Gene Expression Master mix. Total reaction volume was 20 µirwith 300 nM of each primer/probe, 10 µl of master mix, and 1 µl of cDNA as template (or water as a negative control). Primer/probe combinations spanned introns. Beta-Actin primers/probe were: forward 5′-TTGCTGACAGGATGCAGAAG-3′, reverse 5′-TGATCCACATCTGCTGGAAG-3′, probe 5′- TCGGTGGCTCCATCTTGGCC-3′. IL-10 primers/probe were: forward 5′- GAAGACCCTCAGGATGCGG-3′, reverse 5′- ACCTGCTCCACTGCCTTGCT-3′, and probe 5′- TGAGGCGCTGTCATCGATTTCTCCC-3′. Relative expression was calculated using the 2^−ΔΔCt^ method.

### Collagen quantitation

100 mg pieces of liver tissue were excised from perfused livers and snap frozen in liquid nitrogen. Samples were digested in a 0.1mg/ml acid pepsin solution overnight at 4°C, after which. collagen was concentrated and measured using the Sircol Collagen Assay kit (Biocolor UK) as per the manufacturer's instructions.

### Statistics

Data were analyzed with the unpaired Student *t* test via Prism 5.0 (GraphPad Software). All data shown represent mean ± SD, and *p* values≤0.05 were considered statistically significant.

## Supporting Information

Figure S1 **Blocking IL-10R during chronic infection results in a reduction in the production of IgE and IgM within the liver.** Chronically infected Balb/c 4get/KN2 mice were injected bi-weekly with rat anti-mouse IL10R antibody or rat isotype control antibody from weeks 12 - 16 of infection. Perfused livers were digested and the purified cells were assayed for total IgE-secreting cells (**A**) or total IgM secreting cells (**B**) by ELISPOT. Data shown are mean plus/minus SD of three wells of pooled samples of 3 - 5 mice per group, * indicates P<0.05. Data are representative of two independent experiments.
(TIF)

Figure S2 **A model describing the sources and roles of IL-10 during chronic schistosomiasis.** During chronic schistosomiasis, IL-10 promotes the development of schistosome egg antigen-specific IgG1-secreting plasma cells that localize to diseased liver tissue. Whether these cells develop outside or within the liver is unclear, but we have detected both IgG^+^ B cells and IgG1 secreting plasma cells within hepatic infiltrates. Antibodies secreted by plasma cells complex with antigen secreted by schistosome eggs trapped within the liver, and become associated with macrophages. Ligation of FcγR by immune complexes promotes macrophages to assume a regulatory/anti-inflammatory role that is characterized by the production of IL-10 and probably additional factors (?) that prevent the development of severe portal hypertension. The source of IL-10 that initiates this process is probably the population of egg antigen-specific Th2 cells that develops early following the onset of egg production (not shown), but it is possible that IL-10 made by macrophages contributes to the maintenance of plasma cell populations as depicted.
(TIF)
