# Supplementary figures and images for: IL-10R Blockade during Chronic Schistosomiasis Mansoni Results in the Loss of B Cells from the Liver and the Development of Severe Pulmonary Disease
Source: PLoS Pathog. 2012 Jan 26;8(1):e1002490. doi: 10.1371/journal.ppat.1002490 (PMC3266936; doi:10.1371/journal.ppat.1002490)

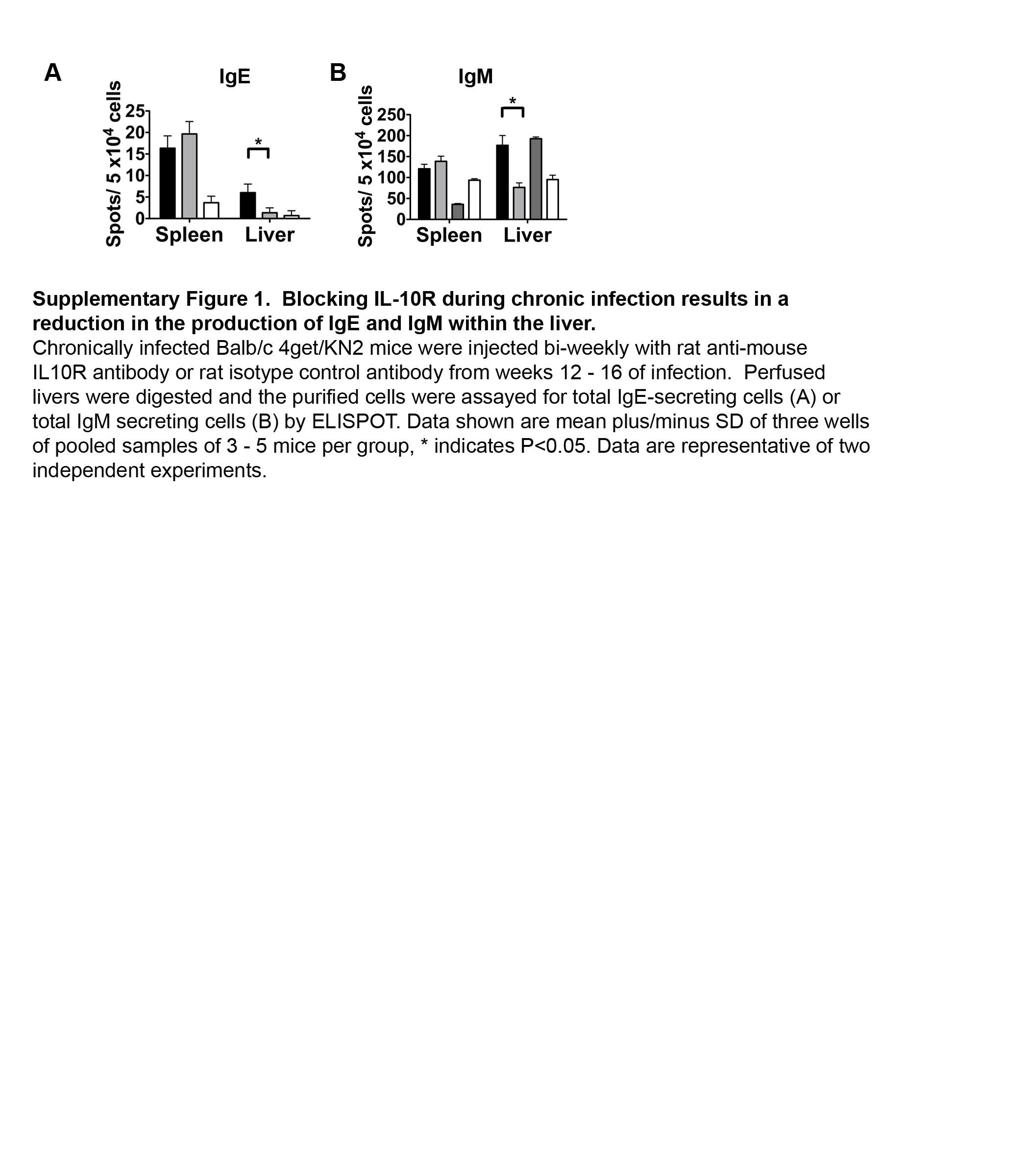

Supplement: Figure S1 — Blocking IL-10R during chronic infection results in a reduction in the production of IgE and IgM within the liver. Chronically infected Balb/c 4get/KN2 mice were injected bi-weekly with rat anti-mouse IL10R antibody or rat isotype control antibody from weeks 12 - 16 of infection. Perfused livers were digested and the purified cells were assayed for total IgE-secreting cells (A) or total IgM secreting cells (B) by ELISPOT. Data shown are mean plus/minus SD of three wells of pooled samples of 3 - 5 mice per group, * indicates P<0.05. Data are representative of two independent experiments. (TIF) [file ppat.1002490.s001.tif]

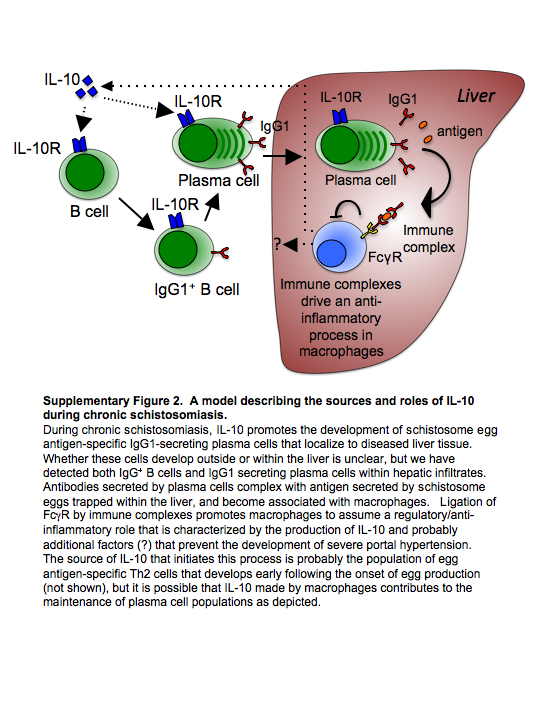

Supplement: Figure S2 — A model describing the sources and roles of IL-10 during chronic schistosomiasis. During chronic schistosomiasis, IL-10 promotes the development of schistosome egg antigen-specific IgG1-secreting plasma cells that localize to diseased liver tissue. Whether these cells develop outside or within the liver is unclear, but we have detected both IgG+ B cells and IgG1 secreting plasma cells within hepatic infiltrates. Antibodies secreted by plasma cells complex with antigen secreted by schistosome eggs trapped within the liver, and become associated with macrophages. Ligation of FcγR by immune complexes promotes macrophages to assume a regulatory/anti-inflammatory role that is characterized by the production of IL-10 and probably additional factors (?) that prevent the development of severe portal hypertension. The source of IL-10 that initiates this process is probably the population of egg antigen-specific Th2 cells that develops early following the onset of egg production (not shown), but it is possible that IL-10 made by macrophages contributes to the maintenance of plasma cell populations as depicted. (TIF) [file ppat.1002490.s002.tif]
